# Spectrum of common Hodgkin lymphoma and non-Hodgkin lymphomas subtypes in Zambia: a 3-year records review

**DOI:** 10.1186/s41043-021-00261-y

**Published:** 2021-08-23

**Authors:** Pascal Polepole, Victor C. Mudenda, Sody M. Munsaka, Luwen Zhang

**Affiliations:** 1 Department of Biomedical Sciences, University of Zambia School of Health Sciences, P.O. BOX 50110, Ridgeway, Lusaka, Zambia; 2 Department of Pathology and Microbiology, University Teaching Hospital, P.BAG RW1X, Lusaka, Zambia; 3 School of Biological Sciences, Nebraska Center for Virology, University of Nebraska, Lincoln, Nebraska 68588 USA

**Keywords:** Spectrum, Lymphoma, Subtypes, Zambia

## Abstract

**Background:**

Lymphomas usually present with different occurrence patterns across different geographical locations, but their epidemiology in Zambia is yet to be extensively explored.

**Objectives:**

To study the spectrum of lymphoma subtypes prevalent within the Zambian population.

**Methods:**

Histopathological records with suspected lymphoma at the University Teaching Hospital (UTH) in Lusaka from the year 2014 to 2016, diagnosed based on the 2008 World Health Organization (WHO) criteria were reviewed. The analysis was done in terms of type, sex, age, and site of biopsy; and Fisher’s exact test was used for significance testing.

**Results:**

During the study period (2014-2016), there were more B cell neoplasms {222 (92.5%)} than T cell neoplasms {18 (7.5%)}. Non-Hodgkin’s lymphoma (NHL) was seen in 191 (79.6%) whereas classic Hodgkin’s lymphoma (CHL) was seen in 39 (16.3%). Burkitt’s lymphoma (BL) and diffuse large B cell lymphoma (DLBCL) showed equal proportions {17.5% of all lymphoma cases (42/240) each}, as the most prevalent subtypes of NHL whereas marginal zone B cell lymphoma was the rarest subtype with 1.4% (4/240). For CHL, mixed cellularity and lymphocyte rich subtypes (4.6% of all lymphoma cases) were the most common subtypes. There was a statistically significant difference in the occurrences of lymphoma subtypes across different age categories (*p* = 0.002).

**Conclusion:**

Zambia has a diverse lymphoma subtypes population, affecting a relatively young population. The data from this study will serve as a baseline for improved health care provision and more robust future studies.

## Background

Lymphoma is a group of blood malignancies that develop from lymphocytes. Also known as lymphoid neoplasms, these blood malignancies comprise a group of closely related yet heterogeneous diseases, with widely varying clinical features, histology, immunophenotypes, and genetic abnormalities [1, 2]. Lymphomas are a fairly common malignancy accounting for approximately half of all newly diagnosed hematological neoplasms, and they comprise the sixth most common group of malignancies worldwide in both men and women, albeit with marked geographic variations and affecting more males than females within the age range of 1 to 85 years but peaking within the second decades of life [3–5]. The highest rates of about 68,000 cases per year are observed in North America while the lowest rates are seen throughout Asia with an incidence rate of around 6.1 [6, 7]. Over the last few decades, the global incidence of lymphoma has recorded an increase of around 3-4% annually [2]. The regions reporting the highest incidence for certain types of lymphomas such as Burkitt’s lymphoma are situated in the developing countries of Africa where poverty levels are still high and the human immunodeficiency virus (HIV) pandemic is still hampering the strides made by health care authorities to reduce disease burdens. The developed countries on the other hand have over the years had a stable increase in the lymphoma incidence [2]. To date, the etiology of most types of lymphoma is not clearly understood [8] but several risk factors have been identified. The most well recognized are acquired immunodeficiency syndrome (AIDS)—infectious agents such as Epstein-Barr virus (EBV), low socio-economic status, advancing age, gender, family history of cancers, dietary, and environmental factors [9–14].

Lymphomas have traditionally been classified as either Hodgkin’s lymphoma (HL) or non-Hodgkin’s lymphoma (NHL) based on the presence or absence of the Reid-Sternberg (RS) cell on histology. HL and NHL are further subtyped into individual entities displaying distinct behavioral, prognostic, and epidemiological characteristics, with varying responses to treatment. NHL subtypes include Burkitt’s lymphoma (BL), diffuse large B cell lymphoma (DLBCL), anaplastic large cell lymphoma, lymphoblastic lymphoma, small lymphocytic lymphoma, and marginal zone B cell lymphoma among others. These make up about 90% of all the lymphoma cases worldwide and have recorded a steady increase in their incidence. By the year 2015, 1 in 78 men and 1 in 110 women at the global level developed NHL between birth and age 79 years. In the same year, NHL was declared the 11th highest cause of cancer deaths [15]. The high prevalence of this cancer in African populations is usually attributed to the high incidence of Burkitt’s lymphoma (BLs) among children in the tropical zone of Africa HL is subtyped into classic Hodgkin’s lymphoma (CHL) and nodular lymphocyte predominance Hodgkin lymphoma (NLPHL). CHL is further subtyped into mixed cellularity, lymphocyte rich, nodular sclerosing, and lymphocyte subtypes [3, 16].

Because of the special geographical location and cultural background in the tropical region of Africa where high levels of poverty, HIV, Epstein-Barr virus, and malaria complicate health care provision, Zambia may have its specific patterns of various cancers. The burden of lymphoma subtypes in Zambia is not clearly known and very few studies have been conducted with pediatric lymphomas being examined for the period 1980 to 1992 [17] and in 2014 [18]. For adult lymphomas, small sample sized studies were carried out [19, 20].

Evidently, all these studies were quite inadequate in scope and this warrants further study. There is need for a holistic investigation of the epidemiology of lymphoma in Zambia, focusing on the subtype, age, sex, and anatomical site. The aim of our study was to determine the overall prevalence of lymphomas and their subtypes at Zambia’s highest referral hospital. The results may establish patterns of lymphoma subtypes currently prevalent in Zambia and will serve as a baseline to carry out more robust studies to design intervention strategies to reduce the burden of lymphomas and other hematological malignancies.

## Methods

### Aim

To study the spectrum of lymphoma subtypes prevalent within the Zambian population.

### Study setting

The study was carried out at the University Teaching Hospital (UTH) in Lusaka, Zambia. UTH is a 2000-bed capacity tertiary hospital and Zambia’s highest referral health institution, and, currently, the final destination of the unsolved medical problems from all over the country [18]. As a highest health institution in the referral system of the country, diseases diagnosed here usually reflect an overall, country wide picture. UTH houses several specialized laboratories including the histopathology laboratory which provide services to most of the country. Also, within the UTH are several training institutions, including the School of Medicine, which is the major provider of medical graduates in the country.

Unfortunately, many medical cases at this institution go undiagnosed only to be discovered at postmortem, mainly due to a lack of prior knowledge about disease burdens, as well as insufficient diagnostic and research infrastructure [21, 22]. Histopathology diagnosis of lymphoma at UTH is based on morphological evaluation of hematoxylin and eosin (H&E)-stained tissues and immunohistochemistry. Flow-cytometry services are insufficient while molecular cytogenetic diagnostic services are not yet available at this institution.

### Study design

**Fig. 1:**
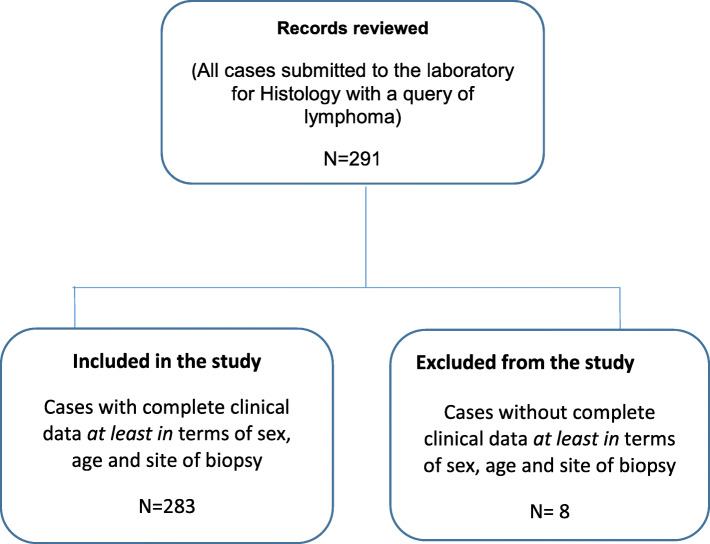
Inclusion and exclusion criteria

This study reviewed all the histopathological records kept over a 3-year period, from 1st January 2014 to 31st December 2016. These cases were submitted to the laboratory as part of a routine diagnostic schedule, with clinical indications or suspicions of lymphoma. The inclusion criteria were all cases with clinical details comprising of at least of sex, age, site of biopsy, and clinical diagnosis or suspicion. All cases that did not meet these criteria were excluded as shown in Fig. 1.

### Statistical analysis

We analyzed all the cases in terms of sex, age, site of biopsy, and final histological diagnosis. Statistical analysis was performed using Stata Version 14 (Stata Corp. 2015) while Fisher’s exact test was used for statistical significance testing and the level of statistical significance was set at *p* ≤0.05. The variables considered were sex, age, and site of biopsy. Sex was categorized as either male or female; age was categorized as different age groups which are as a < 15 years, 15 to 24 years, 25 to 34 years, 35 to 44 years, 45 to 54 years, 55 to 64 years, and 64+ years. Site of biopsy was categorized as either nodal (for the biopsies obtained from lymph nodes) or extra nodal (for biopsies obtained from non-lymph node areas of the body). The frequencies of different lymphoma types and sub-types were stratified by sex and age. Lymphoma classification was done based on the 2008 WHO criteria [23].

## Results

The aim of this study was to find the overall prevalence of lymphoma. During the period considered in this study, a total number of 20,467 biopsies were submitted to UTH histopathology laboratory for routine histological evaluation. The biopsies comprised 6952 for the year 2014, 6428 for the year 2015, and 7087 for the year 2016. Clinical requests querying lymphoma made up 1.4% (291/20467) of the total number of clinical requests to the laboratory in the same period.

**Fig. 2:**
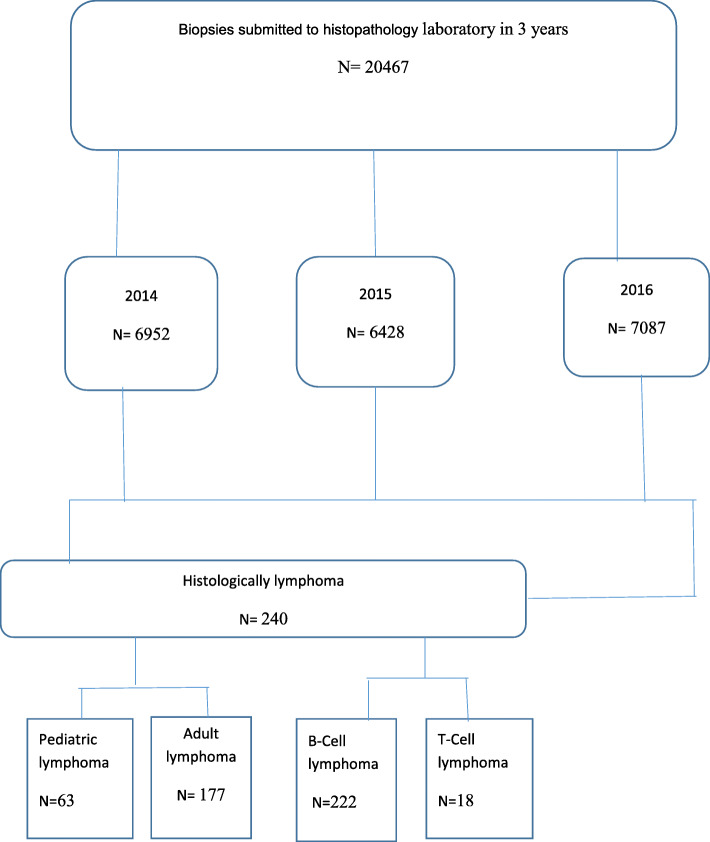
General characteristics of reviewed laboratory records

Two hundred and ninety-one cases of clinically suspected lymphoma were submitted to the histopathology laboratory during the period under review. Out of these, 8 cases were disqualified for incomplete clinical data. Two hundred and eighty-three cases met the study inclusion criteria. After histological typing, 240 (84.8%) had a laboratory diagnosis of lymphoma, 63 (26.2%) in the pediatric age groups and 177 (73.8%) in the adult age groups. B cell lymphoma made up 222 (92.5%) whereas T cell lymphoma made up 18 (7.5%) as shown in Fig. 2. NHL was diagnosed in 191 (79.6%), whereas 39 (16.3%) were diagnosed as the classic type of HL (CHL) and no case of nodular lymphocyte predominance Hodgkin lymphoma (NLPHL) was detected.

Ten (4.1%) of the cases could not be classified as either NHL or HL. The year 2014 recorded the largest submission of cases of clinically suspected lymphoma (108) followed by 2016 (105) while 2015 recorded the lowest (78), but a laboratory diagnosis of lymphoma was only confirmed in 95 cases for the year 2014, 59 for the year 2015, and 86 for the year 2016 as shown in Table 1.

**Table 1:** General demographic characteristics of clinically suspected lymphoma cases at UTH between the year 2014 and 2016 (*N* = 283)

Patients’ characteristics	Lymphoma ***N*** (%)		Not Lymphoma ***N*** (%)
Total submission to the laboratory	240 (84.8%)		43 (15.2%)
	^1^CHL = 39 (16.3)	^2^NHL = 191 (79.6)	
	Unclassified = 10 (4.1%)		
	Not subtyped: CHL = 7 NHL = 71		
**Mean age**	30.6		
**Age groups**			
< 15	63 (26.2)		19 (44.2)
15-24	31 (12.9)		5 (11.6)
25-34	38 (15.8)		6 (14)
35-44	47 (19.6)		4 (9.3)
45-54	34 (14.2)		6 (14)
55-64	16 (6.7)		2 (4.7)
> 64	11 (4.6)		1 (2.3)
**Gender**			
Male	133 (55.4)		28 (65.1)
Female	107 (44.6)		15 (34.9)
**Site**			
Nodal	154 (64.2)		14 (32.6)
Extra-nodal	86 (35.8)		29 (67.3)
**Year**			
2014	95		
2015	59		
2016	86		

^1^Classic Hodgkin’s lymphoma

^2^Non-Hodgkin’s lymphoma

Of the cases diagnosed as lymphoma, 133 (55.4%) were males while 107 (44.6%) were females, setting the overall male:female ratio at 1.2:1. The age of patients with lymphoma ranged from 1 to 84 years, with a mean age of 30.6 years. The peak occurrence of lymphoma was seen among children under 15 years (26.2% of all lymphoma cases) followed by middle-aged patients in the late third and early fourth decade of life (19.6% of all lymphoma cases). Lymphoma was least prevalent among elderly people in the sixth decade (4.6 %). Nodal involvement (64.2%) was more common than extra nodal involvement (35.8%) (Table 1) and the most commonly involved lymph nodes were cervical (34%) while the least involved were epitrochlear (0.68%). The most common extra nodal site of involvement was abdominal (45.2%). Of the 43 cases without a laboratory diagnosis of lymphoma, 28 (65.1 %) were small round blue cell tumor without definitive diagnosis, 11 (25.6%) were carcinoma, while 4 (9.3%) were Castleman’s disease. There was a statistically significant difference in the occurrences of lymphoma subtypes across different age categories (*p* = 0.002) (Table 2).

**Table 2:** Prevalence of lymphoma subtypes by age group at UTH in Lusaka between the year 2014 and 2016 (*N* = 152)

	ALCL^1^	BL^2^	DLBCL^3^	LL^4^	LD^5^	LR^6^	MZCL^7^	MC^8^	NS^9^	SLL^10^	Total
Age groups											
<15	3	19	3	7	0	3	0	5	2	0	42
15-24	1	6	3	1	0	3	0	1	5	1	21
25-34	4	4	9	0	0	2	0	1	3	0	23
35-44	2	7	12	0	1	2	2	2	0	0	28
45-54	3	4	9	0	0	1	1	1	0	2	21
55-64	2	1	3	0	0	0	1	1	0	2	10
> 65	2	1	3	0	0	0	0	0	0	1	7
Total	17	42	42	8	1	11	4	11	10	6	152

*p* = 0.002

^1^Anaplastic large cell lymphoma

^2^Burkitt’s lymphoma

^3^Diffuse large B cell lymphoma

^4^Lymphoblastic lymphoma

^5^Lymphocyte-depleted classic Hodgkin lymphoma

^6^Lymphocyte rich classic Hodgkin lymphoma

^7^Marginal zone cell lymphoma

^8^Mixed cellularity classic Hodgkin lymphoma

^9^Nodular sclerosing classic Hodgkin lymphoma

^10^Small lymphocytic lymphoma

### Non-Hodgkin’s lymphoma

NHL was diagnosed in 191 cases, making up 79.6% of the total lymphoma patients. BL and DLBCL were seen in equal proportions (42/240) as the most prevalent subtypes of NHL, each of them making up 17.5% of all the lymphoma cases. In both subtypes, the majority (60%) of the cases involved lymph nodes. Both subtypes were seen from early childhood to late adulthood but with stark differences in the peak age categories. BL was mainly a disease of patients under 15 years of age while DLBCL was a disease of individuals in their third, fourth, and fifth decades of life (Table 2). BL affected more males than females with a male:female ratio of 1.6 to 1 while DLBCL showed a completely different picture with a male:female ratio of 0.68 to 1. Anaplastic large cell lymphoma made up 7.1% of all lymphoma cases was seen twice as often in lymph nodes as in extra nodal sites and affected more males than females with a male:female ratio of 1.4:1. It had a preponderance for individuals in their third decade of life. Lymphoblastic lymphomas were mainly seen to be a disease of male patients under 15 years of age and diagnosed in the lymph nodes. Small lymphocytic lymphoma and marginal zone B cell lymphoma were both seen as diseases of the middle aged with 2.5% and 1.4% proportions respectively of the total lymphoma cases. Small lymphocytic lymphoma affected males twice as much as females and was largely nodal in location, whereas marginal zone B cell lymphoma affected males and females equally and was seen more in extra-nodal sites (Tables 2 and 3). Seventy-one cases (29.6% of the lymphoma cases) mostly involving lymph nodes were diagnosed as NHL but could not be subtyped further.

**Table 3:** Histological subtype, age range, male to female ratio, frequency, and anatomical location of the lymphoma subtypes diagnosed at the UTH in Lusaka from the year 2014 to 2016 (*N* = 240)

Histological type	Age range (years)	M:F ratio	Frequency		Anatomical location
			(N0)	(%)	(Nodal/extranodal)
Classic Hodgkin’s lymphoma					
Mixed cellularity	6-64	2.7:1	11	4.6	10/1
Lymphocyte rich	9-51	1.5:1	11	4.6	11/0
Nodular sclerosing	12-32	4:1	10	4.2	10/0
Lymphocyte depleted	40		1	0.4	1/0
Not subtyped	6-60	0.2:1	7	2.9	5/2
Non-Hodgkin’s lymphoma					
Burkitt’s lymphoma	1-65	1.6:1	42	17.5	24/18
Diffuse large B cell lymphoma	9-79	0.68:1	42	17.5	24/18
Anaplastic large cell lymphoma	12-71	1.4:1	17	7.1	12/5
Lymphoblastic lymphoma	6-16	2.5:1	7	2.9	4/3
Small lymphocytic lymphoma	19-65	2:1	6	2.5	5/1
Marginal zone B cell lymphoma	41-56	1:1	4	1.4	1/3
Not subtyped	3-78	0.9:1	73	30.4	44/29
Others*	4-84	1:1	10	4.2	3/7
Total			240	84.8	153/87

*Lymphoma cases without histological subtypes

### Classic Hodgkin’s lymphoma

CHL was diagnosed in 39 (16.3%) of the total number of lymphoma patients. Mixed cellularity and lymphocyte rich subtypes were seen in equal proportions as the most common types of CHL, each making up 4.6% of the total lymphoma cases and both had a preponderance of nodal involvement (91% and 100% respectively). Similar age range and peak age category were equally observed among both subtypes with mixed cellularity occurring between 6 and 64 while lymphocyte rich occurred between 9 and 51 years, but both having a peak occurrence within the first and second decades of life. In both subtypes, the male patients were more affected than their female counterparts with a male:female ratio of 2.7:1 for mixed cellularity and 1.5:1 for lymphocyte rich. Nodular sclerosing subtype was prevalent among patients before 40 years of age but mainly affected patients in the 15 to 24 years age category. It made up 4.2% of the total lymphoma cases with a 100% nodal involvement and a male:female ratio of 4:1. Only one 40-year-old male patient had the lymphocyte-depleted subtype of CHL (Tables 2 and 3). Seven cases (2.9%) of all the lymphoma cases were diagnosed as CHL but could not be subtyped further.

## Discussion

Appropriate allocation of resources for cancer prevention, early diagnosis, curative, and palliative care requires detailed knowledge of the local burden of cancer [15]. To the best of our knowledge, our study is the first one to conduct a holistic investigation of the epidemiology of lymphoma in Zambia, focusing on the subtype, age, sex, and anatomical site.

It is a well-established fact that the majority of all lymphoma cases are of B cell lineage [2, 20]. Our study found that the B cell lymphoma accounted for 92.5% of all the lymphoma cases in Zambia, in agreement with most of the findings globally. Our results, however, differ with results obtained from studies done in Kenya, China, Eastern India, and South Korea, which found B cell lymphoma at 80.3%, 76.7%, and 88.8% respectively [1, 24–26]. These variations are not surprising given the geographical trends of lymphoma epidemiology. Even within the same country, ethnic heterogeneity is observed [8, 24, 27]. We found that 79.6% of the lymphoma cases were NHL, and this corroborates the fact that NHL forms a larger proportion of lymphoma diagnosed world over albeit with regional and geographical differences. Our results compare favorably with the Nigerian studies, which found NHL to account for 79.5% and 87.5% of the total lymphoma cases [3, 28]. A representative European study [29] found NHL to be even higher (93.6%) than what is obtaining in sub-Saharan Africa while in Asia, stark variations were observed in the proportions of NHL and HL, but all of the studies agree that NHL comprise over 90% of all lymphoma diagnoses [1, 24, 30]. We contend that variations observed between these studies and ours may be because of varying environmental and genetic factors prevalent in different regions of the world.

In Zambia, only the classical type of HL (CHL) was observed and its subtypes were mainly nodal. It may be deduced that CHL generally affects young people in their first and second decades of life. The absence of nodular lymphocyte predominance Hodgkin lymphoma (NLPHL) in this study corroborates the rarity of this type of HL globally [3, 28, 31–33]. It has been established that the epidemiology of HL exhibits several specific patterns of age-incidence curves, depending on the socioeconomic status of the population [33–35]. Lymphocyte rich and mixed cellularity subtypes of HL were seen more among children under 15 years of age while nodular sclerosing peaked among the young adults, 15 to 24 years old in the Zambian populations. This corroborates the findings that in underdeveloped countries, CHL shows a peak in early childhood with a predominance of mixed cellularity subtype followed by an older adult peak [2]. Epstein-Barr virus (EBV) has traditionally been linked to the etiopathogenesis of HL, especially the mixed cellularity subtype [28, 36]. While the burden of EBV and its involvement in the pathogenesis of lymphoid neoplasms in Zambia has not yet been clearly established, it is noted that the mixed cellularity subtype of CHL is primarily a disease of economically disadvantaged children who are exposed to early childhood viral infection, and older individuals who are immunosuppressed due to old age or HIV infection, while nodular sclerosis is a disease of young adults whose higher social class has delayed their exposure to common childhood infections [34, 37]. This inference is somewhat supported by the findings of one study conducted on herpesviruses among pediatric patients at this site right here in Zambia [38].

Although DLBCL is the most common NHL subtype worldwide, geographical variations exist on a global scale. Most studies in sub-Saharan Africa, including a 1995 pediatric lymphoma study at this same hospital where our study was conducted, found that BLs are the most common subtypes [3, 17, 28, 39]. The situation in sub-Saharan Africa is the opposite of what is obtaining in some regions of Asia, where BL is either present in a small section of the population or non-existent at all [40, 41]. The fact that in our study, BL shares the top notch with DLBCL among the NHL subtypes is not surprising considering our location in the tropical Africa where Malaria is endemic and has been known to exert a cofactor role together with EBV in the etiopathogenesis of BL [9, 42]. However, most studies done at this site found statistics of BL to be very high with some putting it at double the DLCBL proportions. Our study disagrees with these findings and shows equal proportions of these subtypes. The decline in the rates of Burkitt’s lymphoma and the increase in the rates of DLBCL in this region deserve further study. In our opinion, this may be either due to transformation of Lusaka city into a cosmopolitan metropolis or a result of better Malaria control strategies in Zambia that translate into falling incidences of BL. Seven cases of HL and seventy-one cases of NHL could not be subtyped. Failure to subtype these lymphoma cases could be, among other things, a manifestation of insufficient diagnostic infrastructure, which is a common finding in sub-Saharan Africa [3, 43, 44].

The findings of this study should be interpreted in the context of certain limitations. Firstly, while all efforts have been made to present the data as retrieved from the histopathology laboratory records, we feel our findings would have been more robust if we had managed to get more clinical data including the HIV status of the patients. Unfortunately, clinical files were not available at the time of this study. Secondly, while the data presented here is as accurate as when it was used to manage lymphoma patients, misdiagnoses may exist. More robust future research should aim at, among other aspects, establishing the burden of infections among hematological malignancies, as well as locally prevalent genetic and or environmental factors which might be playing a role in the carcinogenesis of hematological malignancies in Zambia.

## Conclusion

Lymphomas in Zambia are mostly of B cell lineage, affecting slightly more males than females and mostly involving lymph nodes. The most common HL subtype is mixed cellularity which is very common among children under 15 years of age while DLBCL and BL present in equal proportions as the most common subtypes of NHL. BL is mainly a lymphoma of both male and female children under 15 years of age while DLBCL is mainly a disease affecting more females than males with its peak among individuals in their fourth and fifth decades of life. Besides the establishment of patterns of lymphoma subtypes currently prevalent in Zambia, this study will serve as a baseline to design intervention strategies to reduce the burden of lymphomas and other hematological malignancies.

## Data Availability

The datasets used during the current study are available from the corresponding author on reasonable request.

## Abbreviations

NHL: Non-Hodgkin’s lymphoma
HL: Hodgkin’s lymphoma
WHO: World Health Organization
BL: Burkitt’s lymphoma
DLBCL: Diffuse large B cell lymphoma
AIDS: Acquired immune deficiency syndrome
HIV: Human immunodeficiency virus
EBV: Epstein-Barr virus
SOM: School of Medicine
CHL: Classic Hodgkin’s lymphoma
UTH: University Teaching Hospital
